# Gene expression changes associated with Barrett's esophagus and Barrett's-associated adenocarcinoma cell lines after acid or bile salt exposure

**DOI:** 10.1186/1471-230X-7-24

**Published:** 2007-06-27

**Authors:** Ying Hao, Sumita Sood, George Triadafilopoulos, Jong Hyeok Kim, Zheng Wang, Peyman Sahbaie, M Bishr Omary, Anson W Lowe

**Affiliations:** 1 Department of Medicine, Stanford University, Stanford, CA, USA; 2 Stanford University Digestive Disease Center, Stanford, CA, USA; 3 Gastroenterology Section, Palo Alto Veterans Affairs Health Care System, Palo Alto, CA, USA

## Abstract

**Background:**

Esophageal reflux and Barrett's esophagus represent two major risk factors for the development of esophageal adenocarcinoma. Previous studies have shown that brief exposure of the Barrett's-associated adenocarcinoma cell line, SEG-1, or primary cultures of Barrett's esophageal tissues to acid or bile results in changes consistent with cell proliferation. In this study, we determined whether similar exposure to acid or bile salts results in gene expression changes that provide insights into malignant transformation.

**Methods:**

Using previously published methods, Barrett's-associated esophageal adenocarcinoma cell lines and primary cultures of Barrett's esophageal tissue were exposed to short pulses of acid or bile salts followed by incubation in culture media at pH 7.4. A genome-wide assessment of gene expression was then determined for the samples using cDNA microarrays. Subsequent analysis evaluated for statistical differences in gene expression with and without treatment.

**Results:**

The SEG-1 cell line showed changes in gene expression that was dependent on the length of exposure to pH 3.5. Further analysis using the Gene Ontology, however, showed that representation by genes associated with cell proliferation is not enhanced by acid exposure. The changes in gene expression also did not involve genes known to be differentially expressed in esophageal adenocarcinoma. Similar experiments using short-term primary cultures of Barrett's esophagus also did not result in detectable changes in gene expression with either acid or bile salt exposure.

**Conclusion:**

Short-term exposure of esophageal adenocarcinoma SEG-1 cells or primary cultures of Barrett's esophagus does not result in gene expression changes that are consistent with enhanced cell proliferation. Thus other model systems are needed that may reflect the impact of acid and bile salt exposure on the esophagus *in vivo*.

## Background

The incidence of esophageal adenocarcinoma has increased at a rate that is among the highest of all cancers[1]. The major risk factor for esophageal adenocarcinoma is the presence of Barrett's esophagus, a premalignant neoplastic lesion that is characterized by intestinal metaplasia replacing the normal squamous esophageal epithelia[2]. The presence of Barrett's esophagus increases the overall risk of adenocarcinoma by 40-fold[3]. In addition, similarities in the gene expression profile of Barrett's esophagus to esophageal adenocarcinoma further support a close relationship between the two tissues[4].

Clinical studies have identified esophageal acid reflux as a risk factor for Barrett's esophagus and adenocarcinoma[3]. *In vitro *experiments using adenocarcionoma and non-neoplastic Barrett's esophageal cell lines established that short-term exposure to an acidic environment results in increase cell proliferation [5-7]. In addition, activation of signal transduction pathways associated with cell proliferation is observed with acid exposure. Primary cultures of Barrett's esophageal tissues exposed to short pulses of acid or bile salts show increased immunocytochemical staining for PCNA or [^3^H]thymidine uptake, which serve as markers for cell proliferation [8-10].

Gene expression profiles based on a genome-wide assessment of gene expression represent a highly sensitive means of determining differences between cells or tissues. There are now numerous examples where gene expression profiles produce novel classifications of tissues in a manner that reflects differences in biological function or clinical outcomes[11,12]. This study addresses whether exposure of Barrett's-associated adenocarcinoma cell lines or primary Barrett's esophageal tissues to acid or bile salts results in differences in gene expression patterns that support transformation to a malignant state. If present, changes in gene expression would provide an opportunity to determine how acid or bile salts contribute to malignant transformation, and may also identify disease-associated markers that are useful for assessing patient risk or as therapeutic targets.

## Methods

### Tissue samples

Unselected patients scheduled for endoscopic evaluation for Barrett's esophagus were enrolled to participate in the study. Biopsies were obtained according to the Seattle protocol using a standard esophagogastroduodenoscope (Olympus GIF-XV10) and biopsy forceps (Radial Jaw 3, Boston Scientific Corp., Natick, MA). Four biopsies each were obtained from normal appearing esophagus (proximal to the Barrett's esophagus), salmon-colored Barrett's esophagus, and the duodenum. Twin biopsies were also obtained for each Barrett's esophagus sample and sent to pathology for analysis. All procedures were performed with patient consent and under approved human subjects protocols from Stanford University and the Palo Alto Veterans Affairs Health Care System.

As previously described, the biopsies were immediately cultured in HEPES buffered Medium 199, pH 7.4, supplemented with 10% heat-inactivated fetal calf serum, 1 ug/ml insulin (Sigma-Aldrich, St. Louis, MO), 500 U/ml penicillin and 250 U/ml streptomycin[8]. Samples were placed on a sterilized stainless grid in 12 well dishes in a sterile sealed jar (Torsion Balance, Clifton, NJ) that was previously perfused with 95% O_2_-5% CO_2 _and incubated at 37°C. Incubation times were derived from previous studies with an assessment of cell viability determined by cellular lactate dehydrogenase release and histology[8,9].

Cell lines were derived from human esophageal adenocarcinomas associated with Barrett's metaplasia (SEG-1 and OE33), a poorly differentiated adenocarcinoma (TE7), and a squamous cell carcinoma (OE21). SEG-1[13] cells (a generous gift from Dr. David Beer, Univ. of Michigan) were grown at 5% CO_2 _in DMEM supplemented with 4.5 g/L glucose, and L-glutamine (Cellgro, Mediatech, Inc., Herndon, VA), penicillin (100 U/ml), streptomycin (100 U/ml), and 10% fetal bovine serum. Experiments were performed with SEG-1 cells within 15 passages after receipt from Dr. Beer. The OE21 and OE33[14] cell lines (European Collection of Cell Cultures, Wiltshire, United Kingdom) and TE7[15] cells (Dr. T.Nishihira, The Second Department of Surgery, Tohoku University School of Medicine, Japan) were grown in RPMI 1640 with 25 mM HEPES, 10% fetal bovine serum, and penicillin and streptomycin (100 U/ml). The passage number of the OE21, OE33, and TE7 cells were not known, but the gene expression profiles of the same cells have been determined and compared to Barrett's esophagus[4].

### Acid and bile salt exposure

Cell lines were first cultured in serum-free media for 48 hours before exposure for various times to media made acidic with the addition of concentrated HCl to pH 3.5. Following acid exposure, normal tissue culture media at pH 7.4 containing 10% fetal bovine serum was added and incubated for an additional 24 hours. For the tissue explants in primary culture, samples were exposed to media at pH 3.5 for 60 minutes followed by incubation at pH 7.4 for 4 hours. A pH 3.5 was previously demonstrated to induce PCNA and villin expression[8].

Exposure to bile salts was achieved through incubation of the cell lines or tissue explants for 1 hour in a mixture of conjugated bile salts designed to resemble what is found *in vivo*, and consisted of 0.54 mM sodium glycocholate (C_26_H_42_NO_6_Na), 0.1 mM sodium taurocholate (C_26_H_44_NO_7_SNa), 0.2 mM glycocholic acid (C_26_H_43_NO_6_), and 0.1 mM sodium taurochenodeoxycholate (C_26_H_44_NNaO_6_S) at pH 7.4[9]. A formulation of conjugated bile salts called "bile salt cocktail" that included conjugated deoxycholic and chenodeoxycholic acids was also applied to the cell lines and was composed of 0.25 mM sodium glycocholic acid, 0.10 mM sodium taurocholic acid, 0.30 mM sodium glycochenodeoxycholic acid, 0.10 mM sodium taurochenodeoxycholic acid, 0.15 mM sodium glycodeoxycholic acid, and 0.05 mM sodium taurodeoxycholic acid. After exposure to bile salts for 1 hour, the cell lines and tissue explants were incubated in regular culture media without bile salts for an additional 24 hours as previously described[9].

### Microarray procedure

Total RNA (20–120 μg) was isolated using Trizol (Invitrogen, Carlsbad, CA). Total RNA from the primary tissue cultures were amplified once (MessageAmp™ II aRNA Amplification Kit Ambion Inc., Austin, TX). A common human reference RNA served as an internal standard (Universal Human Reference RNA, Stratagene Corp., La Jolla, CA).

DNA microarrays containing 41,125 cDNA clones, representing approximately 24,473 unique genes were printed on microscope slides and processed for hybridization as previously described [16-18]. The microarrays were hybridized with sample RNA labeled with Cy5-dUTP and a common human reference RNA labeled with Cy3-dUTP (Amersham Biosciences, Piscataway, NJ)[18]. The arrays were scanned using a GenePix 4000A scanner (Axon Instruments, Molecular Devices Corp., Palo Alto, CA). Detailed protocols can be found at these websites[16,19].

Features containing artifacts or blemishes were flagged for exclusion from further analysis using GenePix Pro 5.0 (Axon Instruments). The raw data was deposited in the Stanford Microarray Database [20,21], where analysis using unsupervised hierarchical clustering was performed[22]. The expression data were visualized using Treeview [22].

Analysis included a hierarchical clustering algorithm that was applied to both the genes and arrays, and a supervised analysis by multi-class comparison using Significance Analysis of Microarrays (SAM) software [22,23]. Interpretation of genes showing differential expression was facilitated by using GoMiner to classify differentially expressed genes in the context of the Gene Ontology (GO database May 2006)[24,25]. Only genes that displayed at least a 3-fold difference in expression between samples were used in the GoMiner analysis. GO categories were considered significant if it contained more than 14 genes and a false discovery rate (FDR) of less than 0.1.

The entire primary dataset is available at the Stanford Microarray Database [20] or the Lowe laboratory website [26].

### Miscellaneous methods

Cell proliferation was evaluated using three approaches: cell counting with a hemocytometer using trypan blue exclusion, [^3^H]thymidine incorporation, or the colorimetric MTT assay (American Type Culture Collection, Manassas, VA) [27]. Thymidine incorporation was achieved using 1 uCi [^3^H]thymidine (Amersham Biosciences, Piscataway, NJ) added to the culture media in each well for the duration of the experiment. The samples were subjected to 3 freeze-thaw cycles followed by collection with a 96 Mach III harvester (Tomtec, Hamden, CT) and 90 × 120 mm glass fiber filters (Filtermat A, PerkinElmer, Wellesley, MA). The filters were dried and then counted with 5 ml of scintillation fluid (Betaplate Scint, PerkinElmer). The incorporated [^3^H]thymidine was assayed with a Wallac MicroBeta^® ^TriLux scintillation reader (PerkinElmer). Immunoblotting was performed according to the method described by Towbin and used 0.2% Tween 20 (Sigma-Aldrich, St. Louis, MO) in the blotting buffer [28,29].

## Results

### Acid effects on Barrett's esophageal adenocarcinoma cell lines

**Figure 1:**
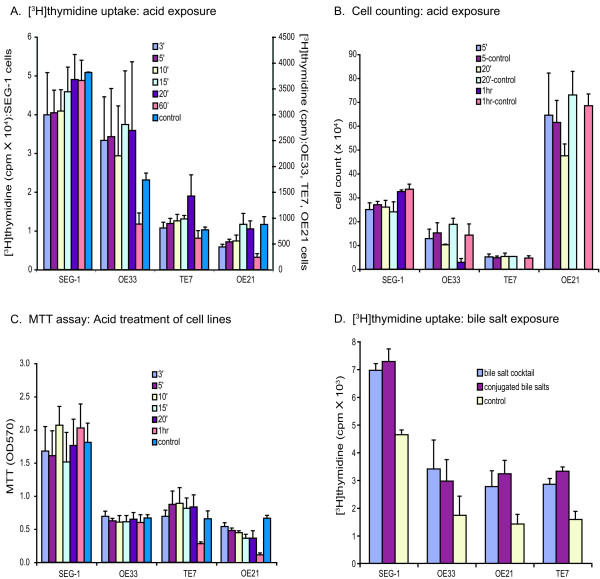
Cell proliferation assays for the esophageal adenocarcinoma (TE-7, SEG-1, OE33) or squamous cell cancer (OE21) cell lines after exposure to acid or bile salts. *Panel A *– [^3^H]thymidine uptake after exposure to pH 3.5 for the indicated times (3, 5, 10, 15, 20, 60 min, and control) followed by a 24 hour incubation at pH 7.4. Each time point is represented by 8 replicates. *Panel B *– Cell counts of cells that exclude trypan blue under the same conditions for acid exposure as panel A. Each time point was assayed in duplicate. Shown is one representative experiment out of 3. *Panel C *– MTT assay for cells treated with acid as described in panel A. *Panel D *– [^3^H]thymidine uptake after exposure to either conjugated bile acids or an cocktail of conjugated bile salts (see Methods) for 1 hr followed by incubation in normal media without bile salts for 24 hours. Each time point is represented by 8 replicates. The error bars represent 1 standard deviation.

Three esophageal adenocarcinoma cell lines (SEG-1, OE33, and TE7) derived from patients with Barrett's esophagus and one squamous esophageal cancer cell line (OE21) were examined for their response to acid incubation at pH 3.5 for 3, 5, 10, 15, 20, and 60 minutes, followed by a 24 hour incubation at pH 7.4. There were no demonstrable changes in cell proliferation as determined by [^3^H]thymidine uptake, cell counting, or the colorimetric MTT assay (Figure 1). An alternative protocol in which serum was not added back to the cells during the incubation at pH 7.4 after acid exposure also did not result in any significant changes using the same assays (data not shown). Incubation in acid for longer than 60 minutes resulted in significant cell death in all the cell lines studied.

With respect to bile salt exposure, both adenocarcinoma and squamous cell carcinoma cell lines showed increased cell proliferation with [^3^H]thymidine incorporation in response to bile salts (Figure 1), but not when evaluated using cell counting or the colorimetric MTT assay (data not shown).

### Gene expression changes associated with acid exposure

**Figure 2:**
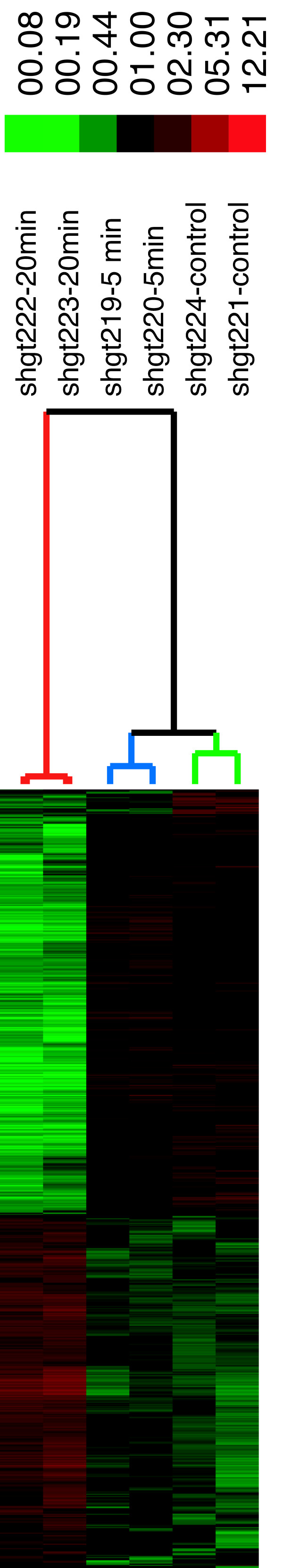
Unsupervised hierarchical clustering of the patterns of variation in expression for 4,218 genes (represented by 4,915 cDNA) in SEG-1 cells at pH 3.5 for 5 or 20 minutes. Each time point was assayed in duplicate. The array dendrogram represents the degree of similarity between arrays. The image uses a color code to represent relative expression levels. Red represents expression levels greater than the mean for a given gene across all samples. Green represents expression levels less than the mean across samples. A color bar (*top*) relates the color code to the magnitude of the differences in gene expression relative to the all-sample mean for each gene. Grey indicates missing or excluded data.

The gene expression profile was determined for SEG-1 cells because of previously published work demonstrating a response to acid[5,6,30,31]. In addition, SEG-1 cells were examined because they possess a gene expression profile most similar to Barrett's esophagus [4]. After acid incubation for 5 or 20 minutes followed by 24 hours incubation at pH 7.4, a genome-wide assessment of gene expression was determined using DNA microarrays. A total of 4,679 genes selected for analysis were differentially expressed at least 3-fold and met filtering criteria for data quality. Supervised and unsupervised approaches for data analysis were used. Application of an unsupervised hierarchical clustering algorithm resulted in the clustering of SEG-1 cells based on the length of acid exposure (Figure 2). In addition, cells with the least amount of acid exposure (5 minutes) were clustered adjacent to the control cells, whereas cells exposed to 20 minutes of acid showed the most dramatic changes in gene expression. A supervised approach of analysis, SAM, that incorporates an assessment of statistical significance to identify differences between cells treated with acid for 20 minutes and untreated cells identified 2,214 genes whose expression was significantly changed at least 3-fold (false discovery rate (FDR) of 0.00027%). Four hundred and thirty-two of these genes showed increase expression and 1,782 genes showed decrease expression compared to controls (see Additional file 1). The differentially expressed genes were also classified in the context of the Gene Ontology to determine whether genes associated with specific biological processes or molecular functions were preferentially represented in response to acid exposure[24,32]. Among those genes expressed at levels lower than controls after acid exposure, no Gene Ontology category was represented with a FDR less than 0.1 (see Additional file 2). For over-expressed genes, the biological process of spermatogenesis (GO:0007283) showed an enrichment of 5-fold (FDR of 0.01), and was represented by 8 genes (Table 1). Genes associated with Gene Ontology terms for cell proliferation (GO:0008283, FDR = 0.9737) or response to wounding (GO:0009611, FDR = 0.6881) did not show a significant change in representation after acid exposure. In contrast, when the same analysis was performed for esophageal adenocarcinoma and Barrett's esophagus tissues based on data from a recently published dataset, genes for cell proliferation were 2.1-fold enriched (GO:0008283, FDR = 0.067) and the response to wounding were 4.9-fold enriched (GO:0009611, FDR < 0.001) (see Additional file 2)[4].

**Table 1:** Upregulated genes in acid treated SEG-1 cells in GO category: Spermatogenesis (GO:0007283)

Symbol	Name
TSPY1	Testis-specific Y-encoded protein 1
SYCP1	Synaptonemal complex protein 1
ADAM28	ADAM metallopeptidase domain 28
TUBD1	Tubulin, delta 1
NME5	Non-metastatic cells 5
DAZL	Deleted in azoospermia-like
TSSK6	Testis-specific serine kinase 6
BRD2	Bromodomain containing 2

**Figure 3:**
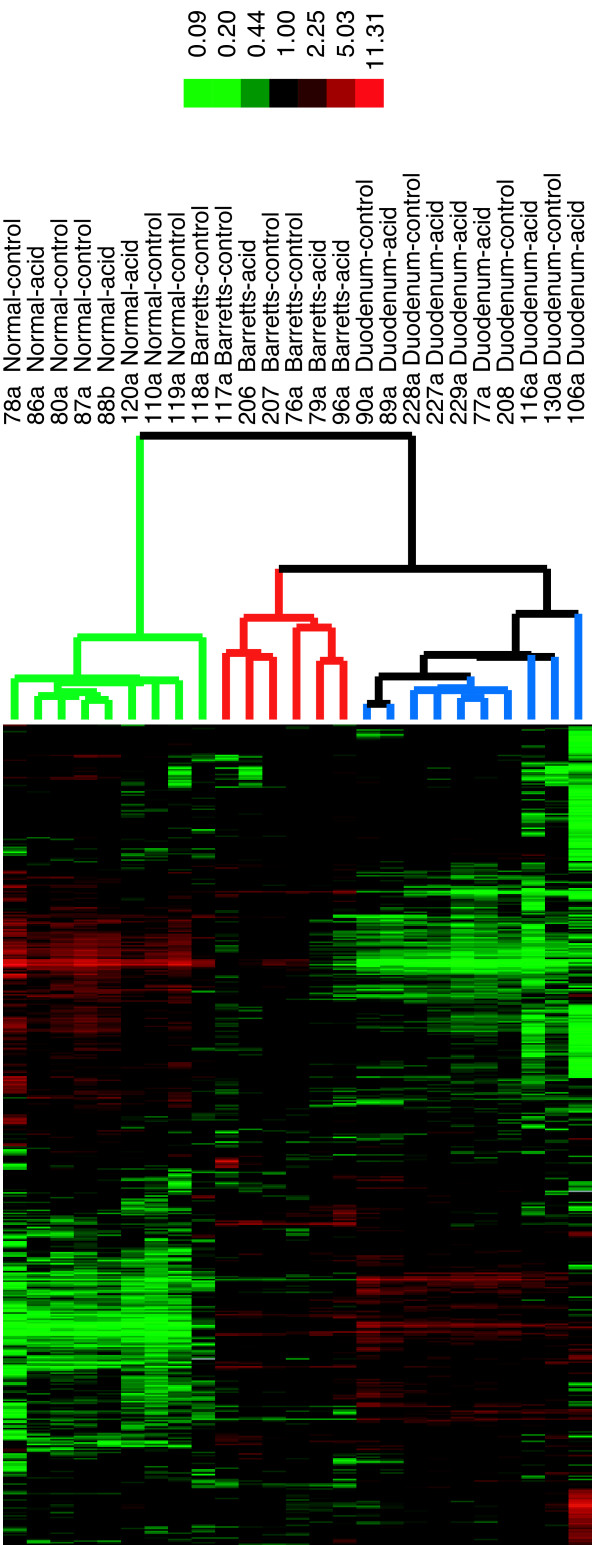
Two-way unsupervised hierarchical clustering of genes (*rows*) and samples (*columns*) based on the patterns of variation in expression for 2,877 genes (represented by 4,318 cDNAs) among 25 tissue specimens derived from 6 patients. Each gene selected for analysis showed at least a 2.8 fold difference in expression from the mean for all samples for at least one array. The image uses a color code to represent relative expression levels. Red represents expression levels greater than the mean for a given gene across all samples. Green represents expression levels less than the mean across samples. A color bar (*top*) relates color code to the magnitude of the differences in gene expression relative to the all-sample means for each gene. Grey indicates missing or excluded data. The dendrogram reflects the relative similarity in gene expression patterns between the arrays.

**Figure 4:**
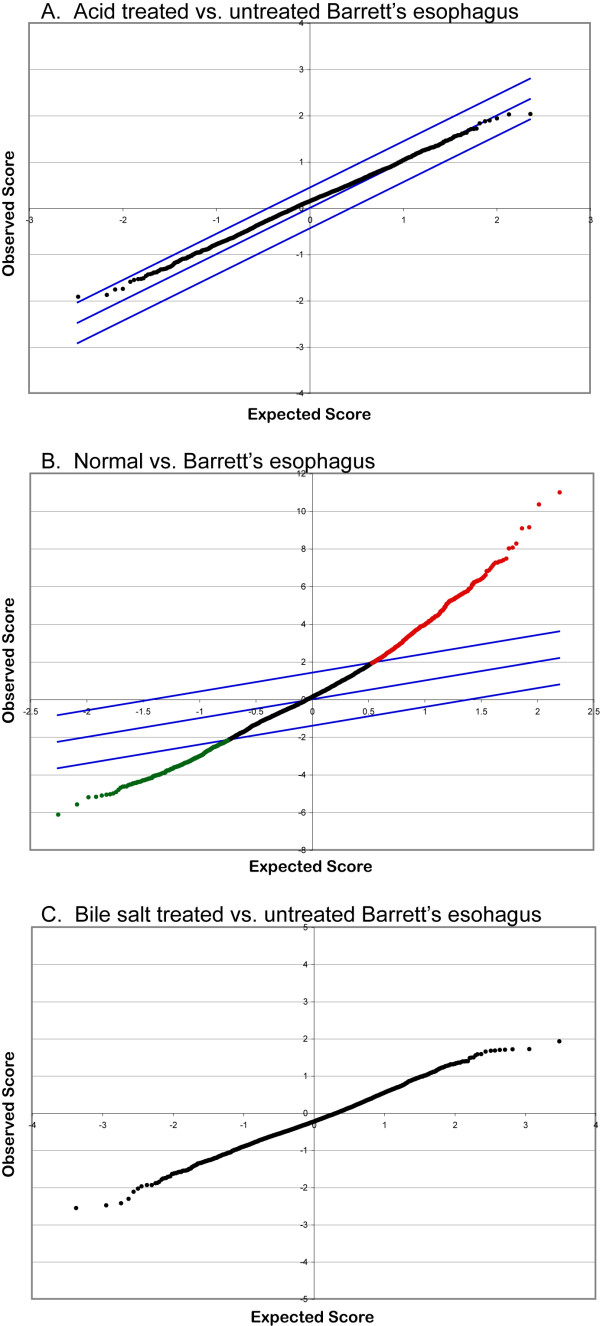
SAM plots of expected to observed values. A: acid treated (3 arrays) versus untreated (5 arrays) primary cultures of Barrett's esophagus (delta = 0.440116) (*top*). B:Normal (8 arrays) versus Barrett's esophagus (5 arrays) primary cultures (delta = 1.410536). Red signifies 545 genes that are overexpressed at least 3 fold and the green represents 258 genes that are underexpressed. C. Barrett's esophagus samples from 3 patients treated with conjugated bile salts versus no treatment.

Primary short-term cultures of Barrett's tissues derived from 6 patients were also examined for changes in gene expression after acid exposure. The 6 patents possessed a history of gastroesophageal reflux for an average of 9 years and had Barrett's esophagus confirmed by pathology. After a 1 hour exposure at pH 3.5, the Barrett's tissues were incubated in media at pH 7.4 for a total of 4 hours before processing for analysis using DNA microarrays. Analysis of the resultant gene expression data using unsupervised hierarchical clustering algorithms produced clusters based on the tissue of origin and not whether there was exposure to acid (Figure 3). Because it is possible that gene expression patterns specific to each tissue are dominant over any changes that may be associated with acid exposure, SAM was used to identify genes that are differentially expressed between acid-treated and untreated Barrett's esophagus tissues. No significant differential expression was detected for any gene (Figure 4). In contrast, the same approach identified 803 genes that are differentially expressed at least 3-fold (FDR = 0.0404) in normal esophagus compared to Barrett's esophagus (Figure 4). Cyclooxygenase-2 (PTGS2) expression was 5.4-fold higher in Barrett's versus normal esophagus tissues, but did not change significantly after acid exposure (see primary dataset).

### Gene expression changes associated with bile exposure in cell lines and Barrett's tissues

Exposure of all cell lines to conjugated bile salts resulted in significant incorporation of [^3^H]thymidine (Figure 1). No changes in cell proliferation as determined by cell counting or the MTT colorimetric assay were observed.

Barrett's tissues from 3 patients were also exposed to a cocktail of conjugated bile salts for 1 hour followed by 24 hours incubation without bile salts[9]. Gene expression analysis using unsupervised hierarchical clustering revealed no evidence of new classifications based on bile salt exposure. Supervised analysis using SAM also did not reveal any differences in gene expression in Barrett's tissues secondary to bile salt exposure (Figure 4).

## Discussion

Our goal was to determine whether acid or bile salt exposure results in gene expression patterns that reflect changes in biological processes within the cell. Previous work in cell lines demonstrated that short exposure to acid results in activation of mitogen-activated protein kinase pathways that contribute to cell proliferation[5]. Work performed in primary Barrett's tissues also showed that only short and not continuous exposure to acid results in increased PCNA expression, a marker of DNA replication, as determined by the number of positive staining cells using immunohistochemistry[8]. Despite using different periods of acid exposure, increased cell proliferation in esophageal adenocarcinoma cell lines was not detected using direct cell counting, colorimetric MTT assays, or [^3^H]thymidine uptake. Changes in PCNA levels as determined by protein immunoblotting also was not detected (data not shown). Changes in PCNA expression, however, may be difficult to detect in cancer cell lines like SEG-1 because they are already selected for optimal growth in culture. Bile salt exposure resulted in increase [^3^H]thymidine uptake in all adenocarcinoma and squamous cell cancer cell lines, suggesting that the effects may be non-specific with respect to cell type.

DNA microarrays provide a highly sensitive approach for detecting changes in gene expression on a genome-wide scale. Numerous studies have demonstrated how gene expression patterns are able to classify tissues in a manner that carries greater significance with respect to natural history and clinical outcomes than other modalities such as conventional pathology. Our original goal was to determine the gene expression profiles associated with acid and bile salt exposure. Subsequent analysis with the aid of clustering algorithms and other analytical tools would then provide an assessment of which biological pathways and processes are induced. SEG-1 cells showed changes in gene expression patterns that appeared to be related to the length of acid exposure. When the genes affected by acid exposure were analyzed with respect to the Gene Ontology, representation by genes in the GO category for spematogenesis did show enhanced expression in the acid treated SEG-1 cells. Using a recently published dataset, none of the 8 spermatogenesis genes with enhanced expression in acid-treated SEG-1 cells showed similarly enhanced expression in Barrett's esophagus tissues or esophageal adenocarcinomas[4]. In addition, SEG-1 cells exposed to acid did not show enhanced expression for any gene or enhanced representation by any GO category that is similarly affected in esophageal adenocarinoma or Barrett's tissues. Thus the significance of the acid-induced gene expression changes observed in SEG-1 cells is unclear. Whether recently developed cell lines that represent non-neoplastic Barrett's cells will show different gene expression profiles in response to acid or bile salt exposure remains to be determined[30,33,34].

A potential problem with the experiments using SEG-1 cells is that they already represent cancer cells that have undergone malignant transformation and thus may not exhibit the same changes in gene expression with acid exposure as a cell derived from Barrett's epithelia. Thus Barrett's tissues were also examined for changes in gene expression in response to acid and bile salts. No changes in gene expression patterns, however, were observed for primary cultures of Barrett's tissues incubated in acid or bile. Supervised approaches for analysis also did not detect any significant changes in gene expression for any individual gene. As an internal control, each tissue examined (duodenum, normal esophagus, and Barrett's esophagus) displayed gene expression patterns that are consistent with previous work. A potential explanation for the absence of detectable differences in gene expression includes heterogeneity in Barrett's esophageal tissues. Previous studies showed an increase in PCNA staining after acid exposure for only a small percentage of cells[8]. Thus if only a small subset of Barrett's esophageal cells are able to respond to acid or bile exposure, there may not be a significant impact on the overall gene expression profile for the tissue sample analyzed. As PCNA positive cells do not possess any distinct morphological features or known specific cell surface markers, their isolation using selective approaches such as laser capture microdissection is not feasible at the present time. A second potential explanation is that a single exposure to acid or bile salts may be insufficient to induce lasting gene expression changes that are detectable by current technologies. The limiting factor with respect to performing experiments of longer duration with greater exposure to acid or bile salts is the viability of the cell lines or primary cultures in such harsh conditions. For such studies to be successful, experimental models that can withstand conditions similar to those that occur *in vivo *need to be developed.

## Conclusion

In summary, pulse acid or bile exposure of Barrett's esophagus or esophageal adenocarcinoma cell lines do not show expression changes in the same genes that are affected in the transformation to esophageal adenocarcinoma.

## Abbreviations

PCNA – proliferating cell nuclear antigen; SAM – significance analysis of microarrays; FDR – false discovery rate; GO – Gene Ontology

## Competing interests

The author(s) declare that they have no competing interests.

## Authors' contributions

Author Contributions: AWL, GT, MOB, and YH conceived and designed the experiments. YH, SS, J-H K, ZW, and PS performed the experiments. GT and PS provided human specimens. AWL analyzed the data and wrote the paper. AWL, GT, MBO, and YH critically revised the manuscript.

## Pre-publication history

The pre-publication history for this paper can be accessed here:



## Supplementary Material

Additional file 1 Differentially expressed genes of SEG-1 cells with and without acid treatment. List of differentially expressed genes generated by the supervised analysis of SEG-1 cells with and without acid treatment. Analysis was performed using SAM. Click here for file

Additional file 2 Gene ontology categories enriched in acid-treated SEG-1 cells, Barrett's esophagus, and esophageal adenocarcinoma. List of Gene Ontology categories ranked by the relative enrichment of differentially expressed genes represented. Analysis performed for genes differentially expressed among acid-treated and untreated SEG-1 cells (worksheets 2 and 3), and Barrett's esophagus versus esophageal adenocarcinoma tissues (worksheets 4 and 5) derived from ref [4]. Click here for file
